# Association between Maternal Smoking during Pregnancy and Low Birthweight: Effects by Maternal Age

**DOI:** 10.1371/journal.pone.0146241

**Published:** 2016-01-21

**Authors:** Wei Zheng, Kohta Suzuki, Taichiro Tanaka, Moriyasu Kohama, Zentaro Yamagata, The Okinawa Child Health Study Group

**Affiliations:** 1 Department of Social Medicine and Health Education, School of Public Health, Peking University Health Science Center, Beijing, China; 2 Department of Health Sciences, Interdisciplinary Graduate School of Medicine and Engineering, University of Yamanashi, Chuo, Yamanashi, Japan; 3 Department of Social Medicine, Faculty of Medicine, Toho University, 5-21-16 Ohmorinisi, Ohta Ward, Tokyo, Japan; 4 Department of Pediatrics, Okinawa Prefectural Chubu Hospital, Okinawa, Okinawa, Japan; Hamamatsu University School of Medicine, JAPAN

## Abstract

**Background:**

Maternal smoking during pregnancy has been consistently related to low birthweight. However, older mothers, who are already at risk of giving birth to low birthweight infants, might be even more susceptible to the effects of maternal smoking. Therefore, this study aimed to examine the modified association between maternal smoking and low birthweight by maternal age.

**Methods:**

Data were obtained from a questionnaire survey of all mothers of children born between 2004 and 2010 in Okinawa, Japan who underwent medical check-ups at age 3 months. Variables assessed were maternal smoking during pregnancy, maternal age, gestational age, parity, birth year, and complications during pregnancy. Stratified analyses were performed using a logistic regression model.

**Results:**

In total, 92641 participants provided complete information on all variables. Over the 7 years studied, the proportion of mothers smoking during pregnancy decreased from 10.6% to 5.0%, while the prevalence of low birthweight did not change remarkably (around 10%). Maternal smoking was significantly associated with low birthweight in all age groups. The strength of the association increased with maternal age, both in crude and adjusted models.

**Conclusions:**

Consistent with previous studies conducted in Western countries, this study demonstrates that maternal age has a modifying effect on the association between maternal smoking and birthweight. This finding suggests that specific education and health care programs for older smoking mothers are important to improve their foetal growth.

## Introduction

Maternal smoking during pregnancy has long been proposed to be one of the most critical preventable factors that can affect the intrauterine environment. [1,2] Studies carried out in different ethnic groups have consistently revealed that maternal smoking is associated with reduced birthweight and elevated prevalence of low birthweight. [3–5] Moreover, maternal age has also been associated with birthweight. A U-shaped relationship between maternal age and birthweight has been identified; both younger and older mothers are more likely to give birth to low birthweight infants. [6,7] Additionally, a series of physiological changes may occur in older mothers that can make their foetuses more vulnerable to unfavourable environments. [8,9] Therefore, it is plausible that older mothers, who are already at risk of giving birth to low birthweight infants, are more susceptible to the effect of maternal smoking. Identifying these high-risk groups contributes to designing targeted intervention programs. However, regarding the effect of maternal smoking on birthweight, only a few studies have discussed the susceptibility of mothers at different ages. [10, 11, 12] These studies were all carried out in Western countries in children before or around the 1990s. The Japanese population is quite different regarding culture, lifestyle habits, prevalence of smoking, and body mass index (BMI) compared to Western populations. Additionally, there might be some secular changes across different time periods. In addition, parity is another notable factor because of its close relationship to birthweight and maternal age. [13] However, no related previous reports referred to the possible effect of parity. Therefore, in this study, we aimed to examine the association between maternal smoking during pregnancy and birthweight in different age groups by parity in a large sample of a Japanese women conducted between 2004 and 2010.

## Methods

### Study population

The study population was from the Okinawa Child Study, which is a cohort study based on free medical check-ups for children. [14] According to Japanese law, free medical check-ups are provided for the maintenance of children’s health. [15] The mothers were obligated to take their children for regular medical check-ups and bring along the Mother and Child Health Handbook, which recorded the health examinations during pregnancy and at delivery. [16] A questionnaire survey was given to all mothers during the medical check-ups. This study covered more than 82% of infants born in Okinawa during the study period. This study included mothers with singleton pregnancies and their infants born between 2004 and 2010 in Okinawa, Japan.

### Measurements

Maternal and birth characteristics were obtained from related birth records and the questionnaire survey given to the mothers when the infants were 3 months of age. During the survey, the mothers were allowed to refer to their Mother and Child Health Handbooks for related information. Maternal smoking was determined by the question: “Did you smoke during pregnancy?” The answer was “Yes” or “No”. Birthweight was classified into two categories: Low birthweight (<2500 g) and not low birthweight (≥2500 g). Other information investigated included: maternal age at delivery (age accurate to year was categorised into 6 groups (≤19y, 20–24 y, 25–29 y, 30–34 y, 35–39 y, and ≥40 y) for stratified analysis), complications during pregnancy (including anaemia, pregnancy-induced hypertension [or preeclampsia], gestational diabetes, etc.), parity (categorical: 1st, 2nd, and 3rd, or more), birth year (accurate to year), and gestational age (accurate to week).

### Ethics statement

The study was approved by the ethical review board of the University of Yamanashi, School of Medicine and was conducted in accordance with the Guidelines Concerning Epidemiological Research (Ministry of Education, Culture, Sports, Science and Technology and Ministry of Health, Labour and Welfare, Japan). We did not obtain informed consent because the Japanese guidelines permit the use of medical examination data without consent if the data are anonymous. In this study, participants’ information was anonymised prior to analysis.

### Statistical methods

The association between maternal smoking during pregnancy and birthweight was examined using a logistic regression model (for prevalence of low birthweight) and a multiple linear regression model (for birthweight). The association was examined based on stratification by birth year, maternal age alone, and then by maternal age and parity. Potential confounders included in the models stratified by maternal age were gestational age (continuous), parity (categorical: 1st, 2nd, 3rd, or more), birth year (categorical: 2004, 2005, 2006, 2007, 2008, 2009, and 2010), and complications during pregnancy (binominal: yes or no). When stratified by both maternal age and parity, the same potential confounders except for parity were included in the models. All analyses were performed using SAS 9.3 (SAS Institute Inc., Cary, NC, USA).

## Results

Overall, 104415 mothers responded to the questionnaire survey. In total, 92641 (89%) of them completed information on all the characteristics studied and were included in the analysis. Descriptive results of maternal and birth characteristics are shown in Table 1. Mothers had a mean maternal age of approximately 30 years. From 2004 to 2010, the proportion mothers of smoking during pregnancy decreased from 10.6% to 5.0%, while the prevalence of low birthweight (<2500 g) remained stable at a level of approximately 10%. We first examined the association between maternal smoking during pregnancy and low birthweight by birth years (Table 2). The results indicated that in all birth year groups, infants whose mothers smoked during pregnancy were more likely to be of low birthweight compared to infants with non-smoking mothers. The association was stronger in children born between 2008 and 2010 than in children born before 2008.

**Table 1:** Characteristics of participants by birth year.

			Birth year						
			2004	2005	2006	2007	2008	2009	2010
Number of participants			13308	13014	13433	13721	13628	13530	12007
Maternal characteristics									
	Maternal age, years, mean (SD)		29.7 (5.5)	29.8 (5.4)	30.0 (5.5)	30.2 (5.5)	30.2 (5.7)	30.5 (5.7)	30.6 (5.8)
	Complications during pregnancy, yes, n (%)		3359 (25.2)	3169 (24.4)	3402 (25.3)	3664 (26.7)	3588 (26.3)	3778 (27.9)	3243 (27.0)
	Smoking during pregnancy, yes, n (%)		1409 (10.6)	1200 (9.2)	1127 (7.8)	1072 (7.8)	947 (7.0)	879 (6.5)	601 (5.0)
Birth characteristics									
	Birthweight, g, mean (SD)		3003 (434)	3003 (427)	3000 (427)	2996 (431)	3001 (418)	2991 (418)	2994 (410)
	Low birthweight, n (%)		1362 (10.2)	1277 (9.8)	1361 (10.1)	1429 (10.4)	1317 (9.7)	1384 (10.2)	1179 (9.8)
	Gestational age, weeks, mean (SD)		38.4 (4.4)	38.4 (4.2)	38.3 (4.8)	38.3 (4.3)	38.4 (4.0)	38.2 (4.6)	38.0 (5.4)
	Parity								
		1st, n (%)	5716 (43.0)	5463 (42.0)	5669 (42.2)	5569 (42.2)	5533 (40.6)	5536 (40.9)	4911 (40.9)
		2nd, n (%)	4495 (33.8)	4352 (33.4)	4676 (34.1)	4676 (34.1)	4484 (32.9)	4392 (32.5)	3854 (32.1)
		3rd or more, n (%)	3097 (23.3)	3199 (24.6)	3476 (25.3)	3476 (25.3)	3611 (26.5)	3602 (26.6)	3242 (27.0)

SD, standard deviation

**Table 2:** Association between maternal smoking during pregnancy and low birthweight by birth year.

Birth year	Maternal smoking during pregnancy		p for trend	No maternal smoking during pregnancy		p for trend	OR for low birthweight
	N (%)	Low birthweight (%)		N (%)	Low birthweight (%)		
			<0.0001			1	
2004–2005	2609 (9.9)	14.0		23713 (90.1)	9.6		1.53 (1.36–1.73)
2006–2007	2199 (8.1)	14.5		24955 (91.9)	9.9		1.54 (1.36–1.75)
2008–2010	2427 (6.2)	16.3		36738 (93.8)	9.5		1.86 (1.66–2.08)

We subsequently examined the association based on stratification by maternal age and parity, and the results are displayed in Table 3. Teenage mothers had the highest prevalence of smoking during pregnancy, and the prevalence decreased as the mother’s age increased. Additionally, the prevalence of maternal smoking during pregnancy increased with parity. Conversely, the prevalence of low birthweight also differed across maternal age groups. Both younger mothers and older mothers tended to have low birthweight babies. Examination of the associations demonstrated maternal smoking during pregnancy was associated with increased risk of low birthweight in all age groups. The strength of the association increased with maternal age, both in crude and adjusted models (adjusted for gestational age, whether born via caesarean section, parity, birth year, complications during pregnancy). The trends did not change when models were further stratified by parity. The adjusted difference in birthweight between children with non-smoking and smoking mothers increased from 76 g to 160 g with maternal age, and this difference increased with parity.

**Table 3:** Association between maternal smoking during pregnancy and low birthweight by maternal age by parity.

Maternal age (years)		No maternal smoking during pregnancy				Maternal smoking during pregnancy				OR for low birthweight	Adjusted OR for low birthweight	Adjusted difference in birthweight (g) Mean (95% CI)
		N	Proportion (%)	Birthweight (g)	Low birthweight (%)	N	Proportion (%)	Birthweight (g)	Low birthweight (%)			
				Mean (SD)				Mean (SD)				
All children												
	≤19	1331	78.9	2955 (406)	11.5	356	21.1	2900 (451)	16.0	1.47 (1.06–2.04)	1.56 (1.15–2.10)^a^	76 (33–120)^a^
	20–24	11243	87.0	2984 (404)	9.8	1677	13.0	2917 (400)	13.2	1.40 (1.20–1.64)	1.49 (1.29–1.72)^a^	76 (57–95)^a^
	25–29	24099	91.6	3007 (406)	9.0	2211	8.4	2909 (403)	13.3	1.56 (1.37–1.77)	1.65 (1.46–1.87)^a^	108 (91–125)^a^
	30–34	28695	94.0	3015 (422)	9.2	1847	6.1	2900 (441)	14.5	1.66 (1.45–1.91)	1.72 (1.51–1.96)^a^	123 (104–142)^a^
	35–39	16537	94.7	3016 (445)	10.5	934	5.4	2833 (478)	21.1	2.28 (1.93–2.68)	2.35 (2.00–2.77)^a^	189 (160–217)^a^
	≥40	3242	94.7	2997 (480)	12.3	181	5.3	2832 (532)	22.1	2.02 (1.40–2.91)	2.18 (1.53–3.12)^a^	160 (94–228)^a^
1st child												
	≤24	8649	87.7	2973 (398)	10.3	1211	12.3	2919 (419)	13.9	1.41 (1.18–1.68)	1.48 (1.26–1.74)^b^	69 (47–90)^b^
	25–29	11729	93.8	2982 (398)	9.8	773	6.2	2900 (390)	12.7	1.34 (1.08–1.67)	1.50 (1.22–1.83)^b^	99 (72–126)^b^
	30–34	10019	94.9	2974 (423)	10.6	544	5.2	2910 (435)	14.2	1.39 (1.08–1.78)	1.44 (1.13–1.84)^b^	70 (35–105)^b^
	≥35	5194	94.9	2951 (452)	13.6	278	5.1	2808 (451)	21.9	1.78 (1.33–2.40)	1.92 (1.43–2.59)^b^	141 (88–194)^b^
2nd child												
	≤24	3373	84.3	3000 (410)	8.9	628	15.7	2934 (386)	12.4	1.45 (1.11–1.89)	1.75 (1.37–2.23)^b^	81 (48–113)^b^
	25–29	8149	91.6	3023 (407)	8.1	748	8.4	2924 (418)	11.6	1.50 (1.18–1.90)	1.50 (1.20–1.88)^b^	95 (66–123)^b^
	30–34	10604	95.1	3022 (420)	8.8	550	4.9	2901 (432)	14.2	1.71 (1.34–2.20)	1.75 (1.37–2.23)^b^	129 (95–163)^b^
	≥35	6372	95.7	3014 (439)	10.1	285	4.3	2850 (464)	19.7	2.17 (1.60–2.94)	2.32 (1.72–3.13)^b^	176 (126–227)^b^
3rd child or more												
	≤24	811	78.4	2990 (455)	11.8	223	21.6	2833 (424)	16.1	1.43 (0.95–2.17)	1.24 (0.82–1.88)^b^	143 (77–207)^b^
	25–29	4221	86.0	3042 (423)	8.5	690	14.1	2903 (401)	15.8	2.02 (1.61–2.55)	2.07 (1.65–2.59)^b^	137 (104–170)^b^
	30–34	8072	91.5	3055 (419)	8.0	753	8.5	2893 (453)	14.9	2.00 (1.61–2.48)	1.95 (1.58–2.41)^b^	156 (126–186)^b^
	≥35	8213	93.7	3052 (455)	9.6	552	6.3	2836 (515)	21.7	2.63 (2.12–3.26)	2.56 (2.07–3.17)^b^	208 (171–246)^b^

^a^ Adjusted for gestational age, parity, birth year, complications during pregnancy.

^b^ Adjusted for gestational age, birth year, complications during pregnancy.

OR, odds ratio

## Discussion

The study covered 92641 mothers and their infants born between 2004 and 2010 in Okinawa, Japan. A decrease in prevalence of smoking but not in low birthweight was observed during the investigation period. In each period, maternal smoking during pregnancy was associated with increased risk of low birthweight. Because the effect of maternal smoking during pregnancy on birthweight was largest in the most recent period, the prevention of maternal smoking during pregnancy might be a higher priority than previously. Additionally, the strength of the association increased with increasing maternal age.

This study confirmed the association between maternal smoking during pregnancy and risk of low birthweight in mothers from all age groups in a large Japanese population. This result is consistent with many previous studies. [3, 4, 5, 17] The proportion of low birthweight infants born to teenage mothers was higher than that in mothers aged 20–29 years, but the odds ratio of infants being low birthweight in smoking mothers were similar for both age groups. These results indicated that teenage mothers who smoked were not at higher risk for having low birthweight infants than other age groups.

Although numerous studies have discussed the association between maternal smoking during pregnancy and adverse birth outcomes, few have reported on the modifying effect by other unfavourable factors. A few studies reported on the modifying effect of maternal age, and their results were consistent with those yielded by our study. One study that evaluated the outcomes of American infants born between 1984 and 1988 and their mothers indicated that the mean difference in birthweight associated with maternal smoking increased from 117 g to 376 g with maternal age. [10] Another study evaluating American infants born between 1989 and 1994 and their mothers investigated the effect of environmental tobacco smoke on birth outcomes, and its results showed an association between environmental tobacco smoke exposure and the occurrence of low birthweight in older non-smoking mothers, but not in younger non-smoking mothers. [11] A study conducted in mothers who gave birth in Norway between 1970 and 1991 demonstrated that the mean birthweight difference between smoking and non-smoking mothers increased with maternal age from 182 g to 232 g. [12] Because the aforementioned studies were carried out in Western countries and they assessed the outcomes of infants born decades ago, the basic information regarding birth weight and maternal smoking during pregnancy was quite different from that in the present study in Japanese women. The birthweight of the infants and the proportion of maternal smoking during pregnancy were much lower in the present study in Japan, and birthweight associated with maternal smoking was also much lower in this study (76–189 g). Possible reasons for this disparity may include differences in the number of cigarettes used during pregnancy and BMI. The smoking prevalence in the aforementioned studies ranged from 22–27%, much higher than the 5.0–10.6% in this study. The number of cigarettes consumed was not reported in these studies. However, a marked decrease in cigarette pack-years and serum cotinine concentrations during recent decades was observed. [18, 19] Therefore, it is possible that the number of cigarettes smoked was also lower in this study compared with that in the aforementioned studies. Additionally, the average birth weight ranged 3187–3602 g in the aforementioned studies, which was also higher than that in our study (2994–3003 g).

There are several possible explanations for the modified effect in older mothers. First, advanced maternal age is likely to be related to a series of unfavourable conditions for foetal growth. Oocytes and embryos from older mothers are more vulnerable to harmful environments. [8] Poorer placental perfusion and impaired transplacental flux of nutrients have been associated with increased maternal age. [20] These physical changes with age make older mothers more susceptible to harmful factors. Maternal smoking is likely to be one of the important factors. Moreover, older mothers are likely to have a long-term smoking history. A review by Cooper et al. indicated that cumulative exposure might influence oocyte quality [21]. Alternatively, maternal age might be a marker of other unmeasured factors associated with increased risk of low birthweight in smoking mothers, such as maternal BMI. Lower maternal BMI is strongly associated with low birthweight. [22] Meanwhile, BMI seems to be lower in smokers, and the magnitude of the difference between smokers and non-smokers becomes larger with age. [23] It is plausible that augmented differences in BMI by smoking in older age groups accounts partly for the modifying effect of age. However, the study by Ahluwalia et al. indicated that the modifying effect of age was significant after adjusting for maternal BMI and gestational weight gain [11]. Additionally, another study conducted in Japan indicated that prevalence of both overweight and underweight pregnant mothers was higher in smoking mothers [24]. These findings suggest that the modifying effect of age cannot be completely explained by differences in maternal BMI. Education level, socioeconomic status (SES), sleep duration, and life habits, are some other factors that have been associated with birth outcomes and maternal smoking, and these can vary according to maternal age. [25, 26, 27]

In consideration of the close relation between maternal age and parity, we conducted a stratified analysis by parity in this study. The results indicated that increased parity amplified the effect of maternal smoking. This is the first study to report that parity modified the association between maternal smoking and birthweight. The mechanisms related to this modifying effect are not clear. One possible explanation is related to the inverse relation between maternal SES and parity due to reduced fertility in high SES mothers. [28] Low SES may be associated with other harmful factors concurrent with maternal smoking during pregnancy. Previously reported factors include behavioural factors, psychological distress, and biological factors including genital tract infection and inflammation and pathological placental changes. These factors may increase the adverse effect on birth outcomes of maternal smoking during pregnancy. [29]

Some limitations of this study needed to be addressed. First, some confounding factors were not investigated. As mentioned above, maternal BMI, SES, and lifestyles might be different in smoking and non-smoking mothers, and these differences may contribute to the differences in the birthweight of their infants. Lack of information on the above-mentioned factors may exaggerate the modifying effect of age. Second, there may be biases related to the collection of information using a questionnaire survey, especially when information was collected retrospectively. Although the mothers could refer to the Mother and Child Health Handbook during the survey, which recorded the health exams during pregnancy and at delivery, it was not possible to guarantee the accuracy of the reported information. Further, information on maternal smoking during pregnancy was not recorded in the handbook. Self-reported information tends to underestimate the proportion of mothers smoking during pregnancy and might thereby exaggerate or understate the association between maternal smoking and birthweight. However, a similar sensitivity of self-reported smoking in mothers aged 25–34 years and mothers older than 35 years was observed by Kvalvik et al. [30]

In conclusion, this study confirmed the increased risk of giving birth to low birthweight infants in Japanese mothers with smoking habits during pregnancy and indicated that age had a modifying effect on this association, although it is unclear on the basis of the current evidence whether the effect was caused by age or by other potential age-related factors. However, the consistent difference across age groups in various ethnic populations and time periods indicates the need for intervention. No matter what the mechanism is, it is necessary to pay special attention to older mothers with smoking habits when carrying out education programs. Additionally, special perinatal care may be essential for older smoking mothers because of the higher proportion of foetal growth restriction in their infants.
